# Effect of three years’ seasonal malaria chemoprevention on molecular markers of resistance of *Plasmodium falciparum* to sulfadoxine-pyrimethamine and amodiaquine in Ouelessebougou, Mali

**DOI:** 10.1186/s12936-022-04059-z

**Published:** 2022-02-08

**Authors:** Almahamoudou Mahamar, Kelsey M. Sumner, Brandt Levitt, Betsy Freedman, Aliou Traore, Amadou Barry, Djibrilla Issiaka, Adama B. Dembele, Moussa B. Kanoute, Oumar Attaher, Boubacar N. Diarra, Issaka Sagara, Abdoulaye Djimde, Patrick E. Duffy, Michal Fried, Steve M. Taylor, Alassane Dicko

**Affiliations:** 1 Malaria Research & Training Center, Faculty of Medicine, Pharmacy and Dentistry, University of Science, Techniques and Technologies (USTT), Bamako, Mali; 2 Division of Infectious Diseases, Duke University Medical Center, Durham, NC USA; 3 Laboratory of Malaria Immunology and Vaccinology (LMIV), National Institute of Allergy and Infectious Diseases (NIAID), National Institutes of Health (NIH), Bethesda, MD USA; 4 Centre de Santé de Référence de Ouelessebougou, Koulikoro, Mali; 5 Department of Epidemiology, UNC Gillings School of Global Public Health, Chapel Hill, NC USA; 6 Duke Global Health Institute, Duke University, Durham, NC USA

**Keywords:** Seasonal malaria chemoprevention, *Plasmodium falciparum*, Molecular markers of resistance, Sulfadoxine-pyrimethamine, Amodiaquine

## Abstract

**Background:**

In 2012, seasonal malaria chemoprevention (SMC) was recommended as policy for malaria control by the World Health Organization (WHO) in areas of highly seasonal malaria transmission across the Sahel sub-region in Africa along with monitoring of drug resistance. We assessed the long-term impact of SMC on *Plasmodium falciparum* resistance to sulfadoxine-pyrimethamine (SP) and amodiaquine (AQ) over a 3-year period of SMC implementation in the health district of Ouelessebougou, Mali.

**Methods:**

In 8 randomly selected sub-districts of Ouelessebougou, Mali, children aged 0–5 years were randomly selected during cross-sectional surveys at baseline (August 2014) and 1, 2 and 3 years post-SMC, at the beginning and end of the malaria transmission season. Blood smears and blood spots on filter paper were obtained and frequencies of mutation in *P. falciparum* genes related to resistance to SP and AQ (*Pfdhfr*, *Pfdhps*, *Pfmdr1*, and *Pfcrt*) were assessed by PCR amplification on individual samples and PCR amplification followed by deep sequencing on pooled (by site and year) samples.

**Results:**

At each survey, approximately 50–100 individual samples were analysed by PCR amplification and a total of 1,164 samples were analysed by deep sequencing with an average read depth of 18,018–36,918 after pooling by site and year. Most molecular markers of resistance did not increase in frequency over the period of study (2014–2016). After 3 years of SMC, the frequencies of *Pfdhps* 540E, *Pfdhps* 437G and *Pfcrt* K76T remained similar compared to baseline (4.0 *vs* 1.4%, p = 0.41; 74.5 *vs* 64.6%, p = 0.22; 71.3 *vs* 67.4%, p = 0.69). Nearly all samples tested carried *Pfdhfr* 59R, and this proportion remained similar 3 years after SMC implementation (98.8 *vs* 100%, p = 1). The frequency of *Pfmdr1* N86Y increased significantly over time from 5.6% at baseline to 18.6% after 3 years of SMC (p = 0.016). Results of pooled analysis using deep sequencing were consistent with those by individual analysis with standard PCR, but also indicated for the first time the presence of mutations at the *Pfdhps* A581G allele at a frequency of 11.7% after 2 years of SMC, as well as the *Pfdhps* I431V allele at frequencies of 1.6–9.3% following 1 and 2 years of SMC, respectively.

**Conclusion:**

Two and 3 years of SMC implementation were associated with increased frequency of the *Pfmdr1* N86Y mutation but not *Pfdhps* 540E, *Pfdhps* 437G and *Pfcrt* K76T. The first-time detection of the *Pfdhps* haplotype bearing the I431V and A581G mutations in Mali, even at low frequency, warrants further long-term surveillance.

## Background

In 2020, malaria caused an estimated 241 million cases and 627,000 deaths worldwide, with most cases occurring in the WHO African Region (> 90%) [1]. Children under 5 years of age are most affected, representing 77% of all malaria deaths worldwide. In the Sahel sub-region of Africa, most childhood malaria mortality and morbidity occur during the rainy season. Administering effective malaria treatment at monthly intervals during this period has been shown to prevent illness and death from malaria in children. Seasonal malaria chemoprevention (SMC), formerly known as intermittent preventive treatment of malaria in children, is the intermittent administration of full treatment courses of an anti-malarial medicine to children during the malaria season, in areas of highly seasonal transmission. An estimated 25 million children aged 3–59 months could benefit from SMC every year [2] and 62% of eligible children benefited in 2018 [3].

Although the safety and effectiveness of SMC are well-established [4, 5], there have been concerns that long-term use of SMC will increase the spread of sulfadoxine-pyrimethamine (SP)- and amodiaquine (AQ)-resistant parasites. SP-resistant parasites can compromise the drug’s effectiveness as a preventive strategy; mutations such as the *Pfdhps* 540E substitution can render SP ineffective in intermittent preventive treatment in infants [6], and the *Pfdhps* 581G substitution (in the presence of the quintuple *Pfdhfr* and *Pfdhps* SP-resistant mutations) undermines SP use as prevention in pregnant women [7]. WHO advises that the presence of mutations at codons 437 and 540 of *Pfdhps,* along with the triple mutation of *Pfdhfr* (quintuple mutation), significantly predicts SP treatment failure; the *Pfdhps* 540 mutant is a useful epidemiological marker of the quintuple mutation in Africa [6]. The mutations *Pfcrt* 76T and *Pfmdr1* 86Y are associated with AQ resistance [8].

The *Pfdhps* A581G mutation in the gene encoding *P. falciparum* dihydropteroate synthetase reduces the efficacy of SP preventive therapy in Malawian pregnant women [9] and no impact of SP in intermittent preventive treatment was found in an area of Tanzania where the frequency of this mutation was high [10]. An increased prevalence of the *Pfdhps* I431V mutation from 0% in 2003 to 36% in 2015 was reported in Nigeria [11] suggesting that these mutations are emerging and need to be monitored in this context. Increases in prevalence of this mutation from 3 to 6% in children under 5 years of age and 2 to 8% were also reported in the ACCESS-SMC study [12] between 2016 and 2018, which covered 7 Sahelian countries, including Nigeria and Mali.

Several studies have previously evaluated the impact of SMC during one season on resistance to SP and AQ, showing no significant difference in the frequency of SP resistance markers [4, 5, 13]. Two recently published studies have shown that SMC was still effective in clearing malaria parasitaemia and preventing clinical malaria after 3 years [12, 14]. However, one of these studies was conducted in the context of a large clinical trial assessing the effect of the addition of azithromycin on hospital admissions and deaths [14], while the other study (ACCESS-SMC) was in conducted in the context of implementation through the health system [12].

It is unknown whether longer periods of SMC use will accelerate the accumulation of resistant parasites, nor the status of resistance to AQ. Most studies have assessed the effects of SMC with SP plus AQ after just one season, but continuous monitoring is required [15]. This study aimed to assess the impact of SMC on molecular markers of *P. falciparum* resistance to SP and AQ after implementation over three consecutive malaria transmission seasons in Ouelessebougou, Mali.

## Methods

### Study site and intervention

The study was conducted in children aged 3–59 months in the health district of Ouelessebougou (located 80 km south of Bamako, Mali), wherein SMC was implemented progressively across sub-districts. To assess the long-term impact of SMC on resistance to SP and AQ, 8 sub-districts were randomly selected in 2014 from the 13 sub-districts of Ouelessebougou to receive SMC over a period of 3 years: 4 sub-districts in 2014 (year 1); 2 sub-districts in 2015 (year 2); and 2 sub-districts in 2016 (year 3). The larger number of sub-districts in year 1 was justified by the need to cover a larger number of villages in year 1 to determine the optimal delivery method of the strategy [16]. SMC was implemented in the remaining sub-districts of Ouelessebougou in 2016.

Eligible children received three rounds of SMC in 2014 and four rounds in 2015 and 2016. SMC was given at monthly intervals during the peak of the malaria transmission season, starting in August. During each round, children aged 3–11 months received 75 mg of AQ given once daily for 3 days, plus a single dose of 250/12.5 mg of SP; children aged 12–59 months received 150 mg AQ base given once daily for 3 days and a single dose of 500/25 mg of SP. The single dose of SP was given only on the first day, simultaneously with the first dose of AQ. Children were observed for 30 min after drug administration, and the medicine was re-administered if vomiting occurred during this period.

### Cross-sectional surveys and sample collection

From 2014 to 2016, at the beginning and the end of each malaria transmission season, a cross-sectional survey was conducted in a random sample of children to assess the prevalence of malaria infection and molecular markers of resistance to SP and AQ. In 2014, 571 and 581 samples were collected at baseline and at the end of malaria season, respectively; in 2015, 429 and 487 samples were collected at the beginning and the end of malaria transmission season, respectively; and in 2016, 747 and 952 samples, at the beginning and the end of malaria transmission season, respectively. Children were selected using simple random sampling from a census list of eligible children in the study areas, to receive SMC during that year except in December 2016 when only children aged 34–59 months were randomly selected to allow assessment of the impact of SMC on malaria immunity [17]. Selected children were examined, and a blood sample was obtained for analysis of molecular markers via blood smear microscopy and dried blood spots (DBS) on filter paper. DBS were sealed in individual plastic bags with desiccant. Baseline samples were collected at the beginning of the malaria transmission season prior the first round of SMC in 2014.

### Laboratory analyses

#### Light microscopy

Thick blood smears were stained with 10% Giemsa for 15 min and read by certified microscopists. Asexual parasites were counted until 200 white blood cells (WBCs) were seen, and blood parasite densities were calculated assuming 8000 WBC/μL. A blood smear was considered to be negative if no parasites were identified in 100 high-power fields. Slides were read by an experienced microscopist blinded to the treatment allocation. Ten per cent of slides were re-read by a blinded expert reader for quality control.

#### Assessment of molecular markers of *Plasmodium falciparum* resistance to SP and AQ

Parasites detected by light microscopy in samples collected through the cross-sectional surveys were genotyped as markers of *P. falciparum* resistance to SP and AQ using two parallel approaches. The first approach used PCR-restriction fragment length polymorphism (RFLP) of specific loci in *Pfdhfr* 59R, *Pfdhps* 437G, *Pfdhps* 540E, *Pfmdr1* 86Y, and *Pfcrt* 76T in individual parasites, hereafter ‘individual sample analysis’, according to published methods [18, 19]. The second approach used PCR amplification and deep sequencing of gene fragments including multiple loci in the same genes plus *Pfdhfr* 51I, *Pfdhfr* 164L, *Pfdhps* 431 V, and *Pfdhps* 581G in pooled parasites, hereafter ‘pooled sample analysis’. Samples were coded and individuals involved in analysis were blinded to groups/time points of the surveys. Samples were analysed on an Ion Torrent platform as previously described [20].

#### Individual sample analysis

DNA was extracted from selected DBS on filter paper (3 M Whatman) as previously described [14, 18]. Assay of drug resistance markers was performed by nested PCR and/or PCR–RFLP [18, 19]. Drug resistance markers tested for this study were *Pfdhfr* 59R for pyrimethamine, *Pfdhps* 437G and *Pfdhps* 540E for sulfadoxine; *Pfcrt* 76T and *Pfmdr1* 86Y for amodiaquine. Results were classified as wild type, mutant or mixed (when both alleles were present).

#### Pooled sample analysis

gDNA extracts from microscopy-positive samples were pooled by year and by site (sub-district) with equal volumes. From these pooled gDNA extracts, relevant drug-resistance loci were amplified in *Pfcrt*, *Pfmdr1*, *Pfdhfr* and *Pfdhps* using separate reactions of a single-round of PCR (Table 1 for primers), and cleaned, pooled and sequenced libraries on an IonTorrent platform as previously described [20]. Fastq files were processed, quality-filtered, aligned to reference sequences of parasite strain 3D7 using Bowtie2, and assessed at variant loci of interest in Galaxy [21]. The output was the proportion of reads covering each locus of interest that harboured a nucleotide substitution encoding a drug-resistance mutation. The same amplification and read-processing methods were applied to the sub-set of samples that were individually deep-sequenced at *Pfdhps*. Two separate fragments were amplified and sequenced for both *Pfdhfr* and *Pfdhps* owing to length constraints for sequencing reads.

**Table 1:** Primers used for deep sequencing of *Pfdhfr, Pfdhps* and *Pfmdr1*

*Locus*, primer name	Sequence
*Pfdhfr*	
PfdhfrF1	CGATCCGAGCGGTGACACATTTAGAGGTCTAGGAAATAAAGG
PfdhfrR1	CCTCTCTATGGGCAGTCGGTGATTTCTTCCCATAACTACAACATTTTGT
PfdhfrF2	CGATCCGAGCGGTGAACAAAATGTTGTAGTTATGGGAAGAA
PfdhfrR2	CCTCTCTATGGGCAGTCGGTGATTTGATAAACAACGGAACCTCCT
*Pfdhps*	
PfdhpsF1	CGATCCGAGCGGTGAGATGGAGGTATTTTTGTTGAACC
PfdhpsR1	CCTCTCTATGGGCAGTCGGTGATATTGGTTTCGCATCACATTT
PfdhpsF2	CGATCCGAGCGGTGATGCATAAAAGAGGAAATCCACA
PfdhpsR2	CCTCTCTATGGGCAGTCGGTGATACAACATTTTGATCATTCATGC
*Pfmdr1*	
MDR1F	CGATCCGAGCGGTGATCAGGAGGAACATTACCTTTTT
MDR1R	CCTCTCTATGGGCAGTCGGTGATACATAAAGTCAAACGTGCATT

Oligos include template-specific sequence as well as overhang adaptors to allow library preparation of amplicons followed by sequencing on an Ion Torrent platform

Substitutions with frequencies below 1% were considered 0

### Data management and statistical analysis

Individual data were entered and verified using DataFax and exported to Stata (version 14, Houston, TX, USA) for analysis. Samples that were individually genotyped using PCR–RFLP with evidence of mixed infection (wild type and mutant) were categorized as mutant. At the beginning and end of the transmission season, proportions of *Pfdhfr* 59R*, Pfdhps* 437G*, Pfdhps* 540E, *Pfmdr1* 86Y, and *Pfcrt* K76T genotypes were determined and compared using Chi square or Fisher exact tests as appropriate.

## Results

### Study population

Demographic characteristics of the study population and malaria prevalence are summarized in Table 2. There was no difference in gender (p = 0.20) or age distribution (p = 0.48) of children surveyed at different time points, except at the 2016 end-of-transmission survey when older children were selected (p < 0.001). Prevalence of malaria infection was significantly higher in children surveyed at 2014 baseline and the 2016 end-of-transmission (p < 0.001).

**Table 2:** Baseline characteristics of participants at each survey

	2014 (Year 1)		2015 (Year 2)		2016 (Year 3)	
	Beginning^a^ (n = 571)	End (n = 581)	Beginning (n = 429)	End (n = 487)	Beginning (n = 747)	End (n = 952)
Age (months)						
Mean (SD)	31.7 (16.4)	33.3 (16.6)	31.0 (15.9)	35.2 (15.2)	32.6 (15.7)	47.5 (8.1)
Median (min–max)	32 (3–59)	32 (4–64)	31 (3–59)	35 (6–62)	33 (3–59)	47 (34–61)
Gender						
Male (%)	49.9	48.3	52.6	53.4	49.0	53.4
*P. falciparum* prevalence	51.9	23.1	37.3	26.1	26.6	49.3

n, number of subjects

^a^Baseline (no SMC)

### Molecular markers of resistance to SP and AQ using individual sample analysis

The frequencies of molecular markers associated with resistance to SP and AQ at baseline and post-SMC are summarized in Table 3. The frequency of *Pfdhps* K540E mutation was low at baseline (4.0%) and did not vary over time with SMC implementation (p = 0.63). The frequency of *Pfdhps* 437G was significantly lower after 2 years of SMC at the beginning of the season compared to baseline (74.5 to 31.6%, p < 0.001), and increased to a level similar to baseline by the end-of-season survey after 3 years of SMC (74.5 *vs* 64.6%, p = 0.22). Nearly all samples tested carried *Pfdhfr* C59R (98.8%) and this proportion remained similar 2 and 3 years after SMC implementation. The frequency of *Pfmdr1* N86Y increased significantly over time from 5.6% at baseline to 18.6% after 3 years of SMC (p = 0.016), while the frequency of AQ resistance marker *Pfcrt* K76T did not vary significantly (p = 0.27) over time after SMC implementation.

**Table 3:** Frequencies of molecular markers of resistance SP and AQ by individual PCR analysis at baseline and 2 and 3 years post-SMC

	August 2014 Baseline		July 2016 Received 2 years of SMC		Dec 2016 Received 3 years post-SMC				
	n/N	%	n/N	%	n/N	%	p^#^	p^##^	p^###^
Pfdhfr C59R	81/82	98.8	46/49	93.9	52/52	100.0	0.147	1	0.089
Pfdhps A437G	73/98	74.5	18/57	31.6	42/65	64.6	< 0.001	0.219	0.000
Pfdhps K540E	4/99	4.0	2/57	3.5	1/70	1.4	1.000	0.405	0.626
Pfmdr1 N86Y	5/89	5.6	5/51	9.8	11/59	18.6	0.497	0.016	0.050
Pfcrt K76T	62/87	71.3	46/57	80.7	31/46	67.4	0.240	0.693	0.272

N, number of samples analyzed; n, number of mutations detected

p^#^ = baseline versus 2 years of SMC

p^##^ = baseline versus 3 years of SMC

p^###^ = comparison of baseline, 2 years of SMC and 3 years of SMC

Baseline (pre-SMC): children who never received SMC

### Resistance markers by deep sequencing

A total of 1,164 DBS samples from all time points were analysed by deep sequencing; after pooling by site and year; for each pool an average read depth of 18,018 at *Pfdhfr*, 29,789 at *Pfdhps*, and 36,918 at *Pfmdr1* loci was obtained.

**Fig. 1:**
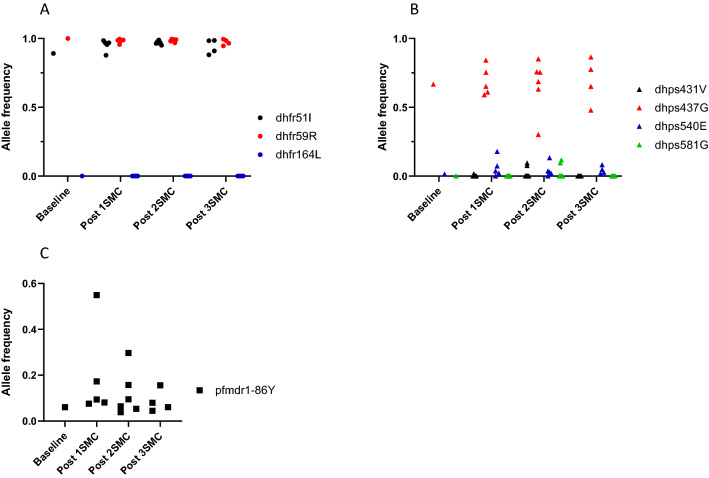
Change in frequency of drug resistance alleles from baseline following 1, 2 or 3 years of SMC administration in pooled parasites by study site. Allele frequencies from the same study site in successive years are presented, with frequencies indicating the proportion of sequencing reads in the year and site that harboured the indicated allele at dhfr (**A**), dhps (**B**), and pfcrt and pfmdr-1 (**C**). Allele frequencies at baseline were estimated from parasites collected prior to SMC implementation in 4 sites and aggregated

At baseline, frequencies of *Pfdhfr* 51I and *Pfdhfr* 59R were vhigh 89.1% and 99.9% respectively and remained high in sites after 1, 2 and 3 years of SMC (Fig. 1). No *Pfdhfr* 164L frequencies exceeding of 1% in any year or site were observed.

The baseline frequency of *Pfdhps* 437G (66.7%) was similar to those after 1, 2 or 3 years of SMC, which ranged from 30.1 to 86.4% without clear evidence of increases over time. *Pfdhps* K540E frequency was low at baseline (1.3%) and remained low following SMC, reaching a highest community frequency of 17.8% 1 year post-SMC introduction. The *Pfdhps* A581G mutation was observed in only two communities at frequencies of 9.6 and 11.7% both after 2 years of SMC. Similarly, the *Pfdhps* I431V allele was observed in only three communities at frequencies of 9.3, 7.5 and 1.6%, after 2 or 1 year of SMC.

In contrast, the low baseline frequency of *Pfmdr1*-86Y allele (6.1%) was exceeded by all sites in most years following SMC, with frequencies ranging from 3.8 to 54.9%. Because measurable frequencies were observed of *Pfdhps* mutations I431V and A581G in separate pools of 2015 and 2016, the 78 individual parasites in these two pools were deep sequenced. Genotyping at each locus was successful in 74 parasites, for which a median depth was observed of 34,796 (*Pfdhps* I431V) and 20,032 (*Pfdhps* A581G) reads. Nine of these (12%) harboured the *Pfdhps* I431V mutation, and 7 of these 9 (78%) also harboured the *Pfdhps* A581G mutation. The frequencies of each mutation were highly correlated within each infection (correlation coefficient = 0.99). Two parasites with the *Pfdhps* I431V mutation lacked the *Pfdhps* A581G allele, and there were no parasites with the *Pfdhps* A581G mutation that lacked the *Pfdhps* I431V mutation.

## Discussion

An updated systematic review to map SP-resistant *P. falciparum* in 294 surveys of infected humans across Africa from 2004 to 2016 has implicated ongoing SP drug pressure, which may in part arise from intermittent preventive treatment of malaria in pregnancy (IPTp) and SMC programmes [22]. Whether implementing SMC over a long period of time increases the frequency of drug resistance markers, thereby decreasing the effectiveness of this strategy, has always been a concern. Several studies have shown a limited impact of SMC on the prevalence of molecular markers of resistance to SP-AQ in children before and after receiving SMC drugs during one season [4, 5, 13]. The current study evaluated the effect of three consecutive seasons of SMC implementation on molecular markers in children using PCR amplification on individual samples, as well as on pools of all samples by year with deep sequencing.

In the individual sample analysis, SMC over three consecutive malaria transmission seasons was not associated with an increase in frequency of *Pfdhps* 540E and *Pfdhps* 437G mutations, which are most commonly used for monitoring resistance of *P. falciparum* to sulfadoxine. The frequency of *Pfdhps* 540E mutation remained far below the 50% threshold recommended for SP in intermittent preventive treatment in children [6]. Nearly all samples (98.8%) carried the *Pfdhfr* C59R mutation at baseline, making this mutation no longer relevant in monitoring resistance to pyrimethamine. These results are consistent with those obtained at other sites in Mali and other countries in the Sahel [12, 14]. In both those studies, as in this study, the frequency of *Pfdhps* 540E mutation remained low in children under 5 years of age who received SMC for 3 years, as well as those in older age groups who did not receive SMC. Both studies showed that SP + AQ was highly effective against clinical malaria or asymptomatic malaria parasitaemia.

These data are consistent with those reported in previous trials in Mali and in Burkina Faso in 2008, with no significant increase in frequency of these markers *versus* the control group after 1 year of SMC implementation [4, 5, 13]. The data support a systematic analysis of national trends in *P. falciparum* resistance to SP in Africa, where the frequency of *Pfdhps* 540E mutation was 3.5% in 2015 in Mali [23]**.** In Senegal, a study indicated that the overall proportion of children carrying parasites with these mutations was lower in SMC areas than in areas where SMC had not been implemented [24].

The frequency of the *Pfmdr1* 86Y mutation, associated with AQ resistance in children carrying *P. falciparum* parasites, was low (5.6%) at baseline but increased significantly after 3 seasons of SMC implementation (to 18.6%). In a trial of SMC with azithromycin [14], the frequency of *Pfmdr1* 86Y increased in Bougouni, Mali from 5 to 11% between 2014 and 2016, while decreasing from 20 to 10% in Hounde, Burkina Faso. In the ACCESS-SMC study [12], the prevalence of this mutation did not increase after 3 years of SMC.

By deep sequencing, *Pfdhfr* mutations at codons N51I and C59R at baseline were close to 100% and remained at similarly high levels after 2 and 3 years of SMC, indicating that these mutations are no longer useful for monitoring SP resistance in the area. Similar frequencies of these mutations were also reported in the ACCESS-SMC study [12] in 7 Sahelian countries where SMC is largely deployed but also in Kenya [25] where resistance to SP was high.

As seen in the ACCESS-SMC study [12], *Pfdhps* I431V and A581G mutations were detected in the current study population that received SMC with SP plus AQ over 3 malaria seasons. The frequencies of mutations in codons A581G and I431V were low (0.0–4.2 for A581G and 0.2–3.2 for I431V) and consistent with reports in Niger [26] and the ACCESS-SMC study [12]. The clinical or parasitological significance of the I431V mutation is unknown, but the A581G mutation, when present along with quintuple SP-resistance mutations on the *Pfdhfr* and *Pfdhps* genes, has been associated with reduced effectiveness of SP for chemoprevention in pregnant women in East Africa, where an increase of *Pfdhps* S436H was reported recently [25]. Its effect in West Africa may be different, given that these data suggest it occurs on a distinct haplotype that harbours the I431V mutation but lacks the K540E mutation. The appearance of this I/K/G *Pfdhps* haplotype across codons I431V, K540E and A581G following SMC implementation suggests selection by SP, although the failure to detect this in any pools the following year may indicate weak selection, as has been the case for other *Pfdhps* resistance haplotypes. Nevertheless, the findings highlight that codons I431V and A581G should be incorporated into routine molecular surveillance of *Pfdhps* loci in West Africa, through which the clinical significance of these mutations can be understood. Further long-term surveillance of molecular markers should become a routine practice in Mali and other countries implementing SMC.

Limitations of this study include the relatively small number of samples analysed individually by PCR. The strengths include deep sequencing genotyping on a large number of samples, 3-year capture of children receiving SMC, and blinded analysis of the samples.

## Conclusion

Two and 3 years of SMC implementation were not associated with increased frequencies of molecular markers of SP and AQ resistance. The detection of the *Pfdhps* haplotype bearing I431V and A581G mutations for the first time in Mali, even at a low frequency, warrants further long-term surveillance.

## Data Availability

The corresponding author had full access to all the data in the study and data are available at request to the corresponding author.

## Abbreviations

AQ: Amodiaquine
CRT: Chloroquine resistance transporter
DBS: Dried blood spots
DHFR: Dihydrofolate reductase
DHPS: Dihydropteroate synthase
DNA: Deoxyribonucleic acid
MDR1: Multidrug resistance 1
PCR: Protein chain reaction
RFLP: Restriction fragment length polymorphism
SMC: Seasonal malaria chemoprevention
SP: Sulfadoxine-pyrimethamine
WBC: White blood cells
